# Differences in the costs and benefits of choosiness may explain variation in cuckoo egg-matching strategy: a reply to Wang and Liang (2023)

**DOI:** 10.1098/rspb.2023.1219

**Published:** 2023-09-13

**Authors:** Jinggang Zhang, Peter Santema, Zixuan Lin, Lixing Yang, Meijun Liu, Jianqiang Li, Wenhong Deng, Bart Kempenaers

**Affiliations:** ^1^ Ministry of Education Key Laboratory for Biodiversity Sciences and Ecological Engineering, College of Life Sciences, Beijing Normal University, Beijing 100875, People's Republic of China; ^2^ Department of Ornithology, Max Planck Institute for Biological Intelligence, Seewiesen 82319, Germany; ^3^ Edward Grey Institute, Department of Zoology, University of Oxford, Oxford OX1 3PS, UK; ^4^ Academy of Forestry Inventory and Planning, National Forestry and Grassland Administration, Beijing 100714, People's Republic of China; ^5^ School of Ecology and Nature Conservation, Beijing Forestry University, Beijing 100083, People's Republic of China

The arms race between avian brood parasites such as common cuckoos *Cuculus canorus* (hereafter ‘cuckoos’) and their hosts forms a classic model of coevolution [1]. An important defence mechanism of hosts is the rejection of the parasitic egg. In many host species, the probability of rejection is higher if the parasitic egg is more different in appearance from the host's own eggs [2,3]. Cuckoos would thus benefit from preferentially parasitizing nests in which the eggs are most similar to their own. However, despite these benefits, several factors may limit the ability of cuckoos to be selective and follow an egg-matching strategy. First, cuckoos also rely on speed and crypsis when laying an egg, because detection by the host might lead to physical attacks and increase the likelihood of egg rejection. Second, cuckoos may be unaware of the appearance of their own eggs. Evidence for the hypothesis that cuckoos use an egg-matching strategy is mixed [4–8], and whether cuckoos choose nests within a given host population based on egg matching remains an open question.

Recently, we provided experimental evidence showing that cuckoos select host nests following an egg-matching strategy in a population of Daurian redstarts *Phoenicurus auroreus* [9]. Daurian redstarts show a distinct egg-colour dimorphism with females laying either blue or pink eggs, whereby the former are more similar to the pale blue eggs of cuckoos [3,9]. We showed that the natural parasitism rate was higher in blue than in pink host clutches, and that cuckoos almost always chose to parasitize a blue clutch when we experimentally presented a dummy clutch of each colour morph adjacent to active redstart nests [9]. In their comment [10], Wang & Liang question our conclusion and criticize the lack of direct video evidence. Here, we address these criticisms.

Regarding the natural observed parasitism rate, Wang & Liang [10] argue that our result probably suffers from survivorship bias. Redstarts laying pink eggs are more likely to reject the cuckoo egg and they reject it more quickly [3,11]. Although we do not have precise data on the latency of egg rejection, we previously found that most rejectors ejected the parasitic egg within 24 h [11]. The observed lower frequency of a cuckoo egg in pink clutches may thus be explained not by cuckoos being selective, but by a higher probability that the cuckoo egg was rejected by the host before we had a chance to detect it. Thus, we agree that the natural parasitism rate may be underestimated more for pink than for blue clutches, and indeed we already made this argument in the original paper [9].

During previous artificial parasitism experiments, we sometimes found the cuckoo egg model that was rejected by the host on the ground near the nest (approx. 15% of the rejected egg models). We, therefore, also checked the ground surrounding the nest during daily nest visits to investigate the possibility that a cuckoo egg had been rejected by the host. However, in our study [9], we never found a cuckoo egg on the ground. Nevertheless, we agree with Wang & Liang that this lack of finding evidence for egg rejection does not exclude the possibility that cuckoo eggs were rejected by the host before detection. Precisely for this reason we conducted the cuckoo choice experiment.

Regarding our experiment, Wang & Liang [10] raised two issues: (i) they suggest that cuckoo parasitism may have been underestimated in pink clutches due to survivorship bias (as for the natural parasitism rate) and (ii) they highlight the lack of direct video evidence for the process of cuckoo choosiness.

Regarding the first point, we checked all three nests (of each experimental triplet) and the surrounding area for the presence of a cuckoo egg every morning and every afternoon, thereby maximizing the chance of detecting the cuckoo egg. Although we cannot exclude the possibility that a cuckoo egg had been ejected from an active nest before detection, and was not found on the ground, this argument does not apply to the dummy nests that were parasitized, unless the redstart host would have ejected the cuckoo egg from one of the dummy clutches, which seems highly unlikely. Considering only the dummy nests (i.e. disregarding experimental triplets in which the active nest had been parasitized), we now test whether the probability of cuckoo parasitism depends on clutch colour. We found 11 instances where a blue dummy clutch had been parasitized compared to only 1 instance where a pink clutch had been parasitized. Cuckoos were thus much more likely to lay an egg in a blue dummy clutch than in a pink dummy clutch (Fisher's exact test: *p* = 0.002), confirming our original conclusion that they select a clutch based on egg colour.

Regarding the second point, we did in fact make video recordings at a subset of triplets of nests during the experiment and recorded cuckoo visits at two of those triplets. On both occasions, the female cuckoo checked multiple nests before laying an egg. In one case (electronic supplementary material, video S1), a female cuckoo first visited the dummy blue-egg nest (right) and removed one egg without parasitizing the nest. About 3 min later, the cuckoo came back and visited the dummy pink-egg nest (middle) without egg removal or laying. Later the same day, a cuckoo egg was found in the active blue-egg nest (left). Although we did not record the cuckoo laying the egg in the nest, we are confident that the cuckoo egg was from the same individual, because there was only one female cuckoo in this subplot of the study area (as discussed in [9]) and the egg had the same appearance as other cuckoo eggs found in this subplot. In the other case (electronic supplementary material, videos S2 and S3), a female cuckoo first visited the active pink-egg nest (top) and removed one egg without parasitizing the nest (electronic supplementary material, video S2). The cuckoo then visited the dummy pink-egg nest (middle), where it laid the parasitic egg and removed a host egg (electronic supplementary material, video S3). These videos provide direct evidence of the cuckoo's nest selection process, although, in the latter case, the cuckoo did not choose the blue-egg nest.

Wang & Liang [10] highlight several studies to argue that cuckoos use a random egg-laying strategy. However, as they mention in their first paragraph, there are also multiple studies in support of the hypothesis that cuckoos use an egg-matching strategy [4–6], and several experimental studies support the hypothesis that cuckoo egg-laying is non-random with respect to nest features other than egg colour. However, Wang & Liang [10] do not mention or discuss these studies. For instance, Wang and colleagues found that cuckoos preferentially parasitize Oriental reed warbler *Acrocephalus orientalis* nests with a smaller number of eggs [12] or with a larger nest size [13].

Given the mixed evidence in the literature, we question Wang & Liang's implicit assumption that there should be a single answer to the question of whether cuckoos selectively parasitize nests based on egg appearance. We argue that cuckoo behaviour is likely to be influenced by the costs and benefits of being selective, which may vary across different cuckoo–host systems. For instance, the benefits of nest selection based on egg colour should be higher (i) when there is more variability in host egg appearance, such that some clutches clearly resemble cuckoo eggs more closely than others, and (ii) when host nest density is high, such that clutches with varying degree of matching are available to a single female cuckoo at a given time. In addition, the costs of being selective are likely to be lower in host species that do not show physical aggression towards cuckoos. In those systems, a female cuckoo may not have to quickly and secretively lay an egg.

In the case of the Daurian redstart, all these conditions are met. (i) Host eggs display a distinct colour dimorphism and cuckoo eggs consistently resemble the blue, but not the pink morph, such that a female cuckoo can be selective even without knowing the appearance of her own eggs. (ii) Redstarts in our population breed at high density and synchronously, such that a female cuckoo typically has multiple potential host nests to choose from. (ii) Unlike in some other host species [14], redstarts do not engage in mobbing or physical attacks against cuckoos. We have never observed aggressive behaviour towards either a real cuckoo or a taxidermic model. Consequently, this host system probably exerts a stronger selective pressure on cuckoos to be choosy compared to other systems. In conclusion, we advocate for further research assessing the conditions that influence nest selection in cuckoos.

## Data Availability

The data are provided in electronic supplementary material [15].

## References

[1] Davies NB. 2000 Cuckoos, cowbirds and other cheats. London, UK: T & AD Poyser.

[2] Yang C et al. 2010 Coevolution in action: disruptive selection on egg colour in an avian brood parasite and its host. PLoS ONE **5**, e10816. (10.1371/journal.pone.0010816) 20520815 PMC2877083

[3] Zhang J, Møller AP, Yan D, Li J, Deng W. 2021 Egg rejection changes with seasonal variation in risk of cuckoo parasitism in Daurian redstarts, *Phoenicurus auroreus*. Anim. Behav. **175**, 193-200. (10.1016/j.anbehav.2021.03.007)

[4] Avilés JM, Stokke BG, Moksnes A, Røskaft E, Asmul M, Møller AP. 2006 Rapid increase in cuckoo egg matching in a recently parasitized reed warbler population. J. Evol. Biol. **19**, 1901-1910. (10.1111/j.1420-9101.2006.01166.x) 17040387

[5] Cherry MI, Bennett ATD, Moskat C. 2007 Do cuckoos choose nests of great reed warblers on the basis of host egg appearance? J. Evol. Biol. **20**, 1218-1222. (10.1111/j.1420-9101.2007.01308.x) 17465931

[6] Honza M, Sulc M, Jelinek V, Pozgayova M, Prochazka P. 2014 Brood parasites lay eggs matching the appearance of host clutches. Proc. R. Soc. B **281**, 20132665. (10.1098/rspb.2013.2665) PMC3843844 24258721

[7] Antonov A, Stokke BG, Fossøy F, Ranke PS, Liang W, Yang C, Moksnes A, Shykoff J, Røskaft E. 2012 Are cuckoos maximizing egg mimicry by selecting host individuals with better matching egg phenotypes? PLoS ONE **7**, e31704. (10.1371/journal.pone.0031704) 22384060 PMC3285637

[8] Yang C, Wang L, Liang W, Møller AP. 2016 Do common cuckoos (*Cuculus canorus*) possess an optimal laying behaviour to match their own egg phenotype to that of their Oriental reed warbler (*Acrocephalus orientalis*) hosts? Biol. J. Linn. Soc. **117**, 422-427. (10.1111/bij.12690)

[9] Zhang J, Santema P, Lin Z, Yang L, Liu M, Li J, Deng W, Kempenaers B. 2023 Experimental evidence that cuckoos choose host nests following an egg matching strategy. Proc. R. Soc. B **290**, 20222094. (10.1098/rspb.2022.2094) PMC9943643 36809803

[10] Wang L, Liang W. 2023 Random egg laying in host nests, rather than egg-matching, explains patterns of cuckoo parasitism: a comment on Zhang *et al.* (2023). Proc. R. Soc. B **290**, 20231018. (10.1098/rspb.2023.1018) PMC10498026 37700656

[11] Zhang J, Santema P, Li J, Feeney WE, Deng W, Kempenaers B. 2022 The mere presence of cuckoos in breeding area alters egg-ejection decisions in Daurian redstarts. Behav. Ecol. **33**, 1153-1160. (10.1093/beheco/arac084)

[12] Wang L, Yang C, He G, Liang W, Møller AP. 2020 Cuckoos use host egg number to choose host nests for parasitism. Proc. R. Soc. B **287**, 20200343. (10.1098/rspb.2020.0343) PMC7341929 32517623

[13] Wang L, He G, Yang C, Møller A, Liang W. 2022 Nest size matters: common cuckoos prefer to parasitize larger nests of Oriental reed warblers. Anim. Cogn. **25**, 589-595. (10.1007/s10071-021-01574-5) 34773170

[14] Zhao H, Luo H, Yan H, He G, Wang L, Liang W. 2022 Fatal mobbing and attack of the common cuckoo by its warbler hosts. Ecol. Evol. **12**, e9649. (10.1002/ece3.9649) 36568870 PMC9772492

[15] Zhang J, Santema P, Lin Z, Yang L, Liu M, Li J, Deng W, Kempenaers B. 2023 Differences in the costs and benefits of choosiness may explain variation in cuckoo egg-matching strategy: a reply to Wang and Liang (2023). Figshare. (10.6084/m9.figshare.c.6823036) PMC10498025 37700659

